# Non-autotrophic methanogens dominate in anaerobic digesters

**DOI:** 10.1038/s41598-017-01752-x

**Published:** 2017-05-04

**Authors:** Atsushi Kouzuma, Maho Tsutsumi, Shun’ichi Ishii, Yoshiyuki Ueno, Takashi Abe, Kazuya Watanabe

**Affiliations:** 1 School of Life Sciences, Tokyo University of Pharmacy and Life Sciences, Hachioji, Tokyo 192-0392 Japan; 2 R&D Center for Submarine Resources, Japan Agency for Marine-Earth Science and Technology (JAMSTEC), Nankoku, Kochi 783-8502 Japan; 3 Kajima Technical Research Institute, Chofu, Tokyo 182-0036 Japan; 4 Graduate School of Science and Technology, Niigata University, Niigata, Niigata 950-2181 Japan

## Abstract

Anaerobic digesters are man-made habitats for fermentative and methanogenic microbes, and are characterized by extremely high concentrations of organics. However, little is known about how microbes adapt to such habitats. In the present study, we report phylogenetic, metagenomic, and metatranscriptomic analyses of microbiomes in thermophilic packed-bed digesters fed acetate as the major substrate, and we have shown that acetoclastic and hydrogenotrophic methanogens that utilize acetate as a carbon source dominate there. Deep sequencing and precise binning of the metagenomes reconstructed complete genomes for two dominant methanogens affiliated with the genera *Methanosarcina* and *Methanothermobacter*, along with 37 draft genomes. The reconstructed *Methanosarcina* genome was almost identical to that of a thermophilic acetoclastic methanogen *Methanosarcina thermophila* TM-1, indicating its cosmopolitan distribution in thermophilic digesters. The reconstructed *Methanothermobacter* (designated as Met2) was closely related to *Methanothermobacter tenebrarum*, a non-autotrophic hydrogenotrophic methanogen that grows in the presence of acetate. Met2 lacks the Cdh complex required for CO_2_ fixation, suggesting that it requires organic molecules, such as acetate, as carbon sources. Although the metagenomic analysis also detected autotrophic methanogens, they were less than 1% in abundance of Met2. These results suggested that non-autotrophic methanogens preferentially grow in anaerobic digesters containing high concentrations of organics.

## Introduction

Methanogenic archaea (methanogens) are ubiquitously present in anaerobic environments, such as digestive tracts, paddy fields, and aquatic sediments, and play an important role in anaerobic degradation of organic matter and the global cycle of carbon^1, 2^. Additionally, they contribute to human society via their ability to produce methane gas in anaerobic digesters^3^.

In methanogenic ecosystems, methane is produced via syntrophic associations between methanogens and anaerobic bacteria, including fermenters and syntrophs^4, 5^. Fermenters and syntrophs degrade organic substances and produce acetate, formate, methanol, CO_2_, and H_2_, which serve as carbon and/or energy sources for acetoclastic, methylotrophic, and hydrogenotrophic methanogenesis^6, 7^. Among them, acetate serves as the key intermediate metabolite, from which methane is produced by syntrophic acetate oxidation (SAO) coupled to hydrogenotrophic methanogenesis, in addition to acetoclastic methanogenesis^8–10^.

Thus far, a number of microbes have been isolated from methanogenic microbial communities, and their genomic and metabolic features have been characterized^11–14^. Furthermore, recent metagenomic and metatranscriptomic studies have provided insights into uncultured members of methanogenic communities^15–21^. For example, Nobu *et al*. reported that anaerobic degradation of terephthalate in a methanogenic bioreactor was supported by complex synergistic networks comprised of many uncultivated microbes, including fermentative, syntrophic, and acetogenic bacteria^16^. These studies demonstrated that the meta-omics are powerful tools for dissecting the dynamics and ecophysiology of microbes involved in methanogenesis and for addressing the comprehensive view of functional microbiomes. However, since population genomes (bin-genomes) and/or metabolic pathways reconstructed in these studies are not complete, it is predicted that there still exists a number of unexplored mechanisms in uncultured microbes that facilitate their growth and survival in methanogenic ecosystems.

Anaerobic digesters are globally used for the treatment of organic wastes and provide favorable habitats for methanogens^22–24^. Among these are thermophilic packed-bed digesters, in which certain groups of methanogens, including those belonging to the families *Methanosarcinaceae* and *Methanobacteriaceae*, are highly enriched in terms of short hydraulic retention times (HRT)^25, 26^. Studies have suggested that the methanogens specifically enriched in biofilms facilitate highly efficient organics degradation and methane production^27, 28^. However, little is known about the ecophysiology of these methanogens, e.g., how they adapt to high organics loading rates (OLR) and achieve efficient methane production in these digesters.

In the present study, we conducted phylogenetic, metagenomic, and metatranscriptomic analyses of microbiomes established in thermophilic packed-bed anaerobic digesters fed acetate as the major substrate, with a particular focus on characterizing the genomic and metabolic features of uncultured methanogens that preferentially grew there. Deep sequencing of metagenomes and precise binning of assembled contigs reconstructed complete and high-quality draft genomes for abundant methanogens and associated bacteria. Furthermore, metatranscriptomics were conducted to reveal transcriptional dynamics of the methanogens in response to shifts in OLR. These findings provide us with new insight into the ecophysiology and *in-situ* metabolism of methanogens that thrive in anaerobic digesters.

## Results and Discussion

### Enrichment of methanogenic consortia in anaerobic digesters

**Figure 1:**
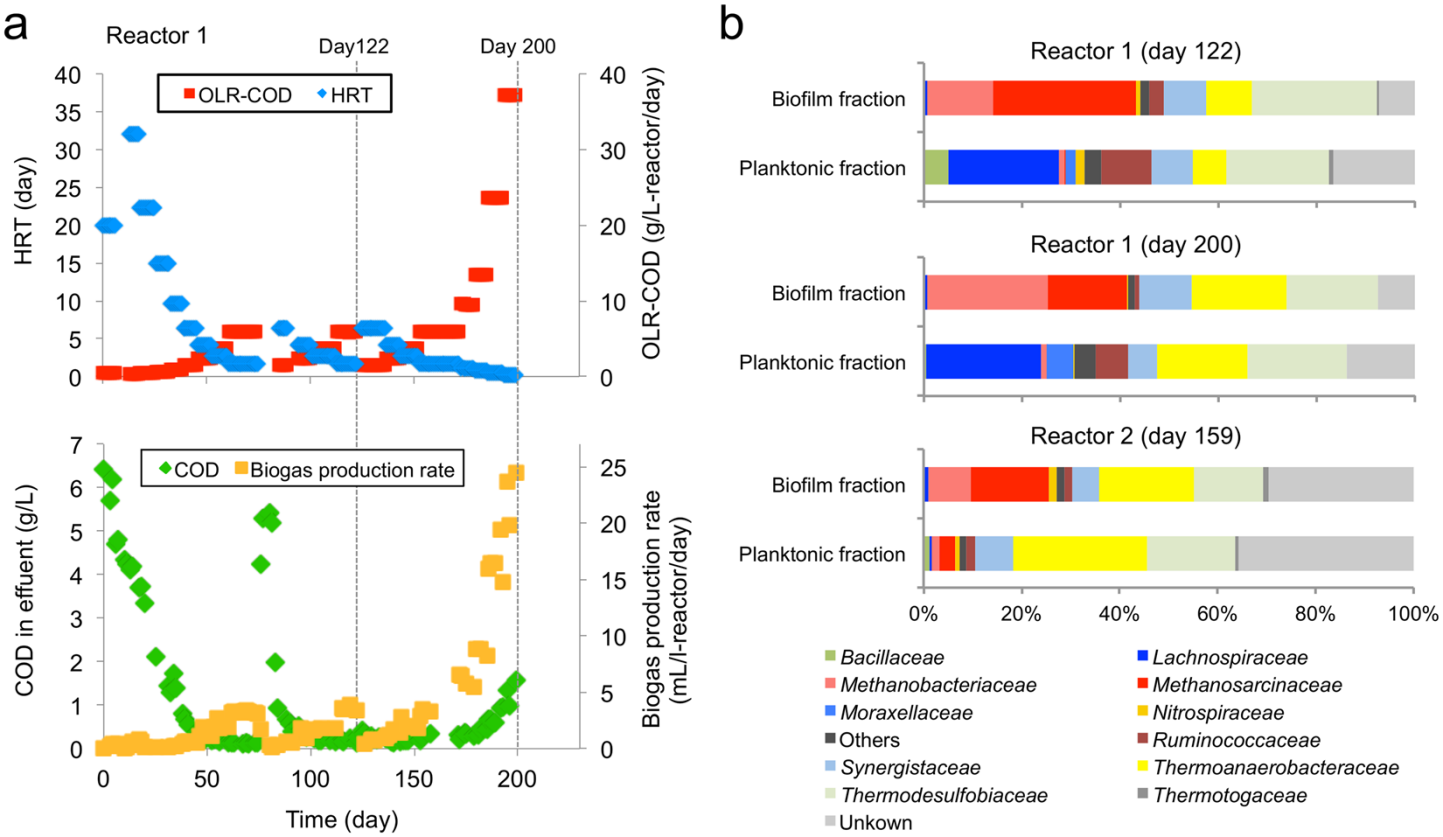
Bioreactor operation and enrichment of methanogenic consortia. (**a**) Time course of OLR, HRT, COD in effluent, and biogas production rates in reactor 1. Data on the operation of reactor 2 are shown in Supplementary Fig. S2. (**b**) Relative abundances of major archaeal and bacterial families in reactor 1 (days 122 and 200) and reactor 2 (day 159) based on pyrosequenced 16S rRNA gene amplicons.

To enrich methanogenic consortia, we operated packed-bed anaerobic digesters using acetate as the major substrate. Two laboratory-scale reactors were operated at 55 °C for 200 days (reactor 1) and 159 days (reactor 2). The schematic diagram of the reactors is shown in Supplementary Fig. S1. Methane was stably produced under high OLR conditions (37.2 g L^−1^ day^−1^ in reactor 1 on day 200; 21.1 g L^−1^ day^−1^ in reactor 2 on day 159; Fig. 1a and Supplementary Fig. S2). Metagenomic DNA and RNA were extracted from biofilm cells attaching onto support media (biofilm fraction; BF) and planktonic cells in fermentation liquid (planktonic fraction; PF) when the operation of these reactors was terminated. To investigate the influence of OLR on methanogenic consortia, samples were also taken from reactor 1 on day 122 (OLR of 5.9 g L^−1^ day^−1^). Data on the performance of the reactors, including organics-removal ratios, methane production yields, and biomass in BFs and PFs, are summarized in Supplementary Table S1. The data indicated that methanogenesis was the major catabolic process in these reactors.

### Phylogenetic analyses of 16S rRNA gene amplicons

PFs and BFs in reactors 1 and 2 were subjected to phylogenetic analyses based on 16S rRNA genes. We found that, in both reactors, methanogens belonging to the families *Methanosarcinaceae* and *Methanobacteriaceae* represented substantial portions (25% to 43%) of the microbiomes in BFs, while these methanogens were only present as minor components (less than 5%) in PFs (Fig. 1b). These results indicate that methanogens were highly enriched in biofilms on support media, contributing to efficient methane production in the packed-bed reactors. The compositions of methanogens are similar to those reported previously for other thermophilic packed-bed reactors^25, 27^, suggesting that members of *Methanosarcinaceae* and *Methanobacteriaceae* are the key methanogens in these digesters. In addition, microbial populations in reactor 1 were not substantially different between day 122 and day 200, suggesting that the major members of methanogens are stably maintained regardless of OLR.

### Phylogenetic analyses of methanogens based on *mcrA* genes

**Figure 2:**
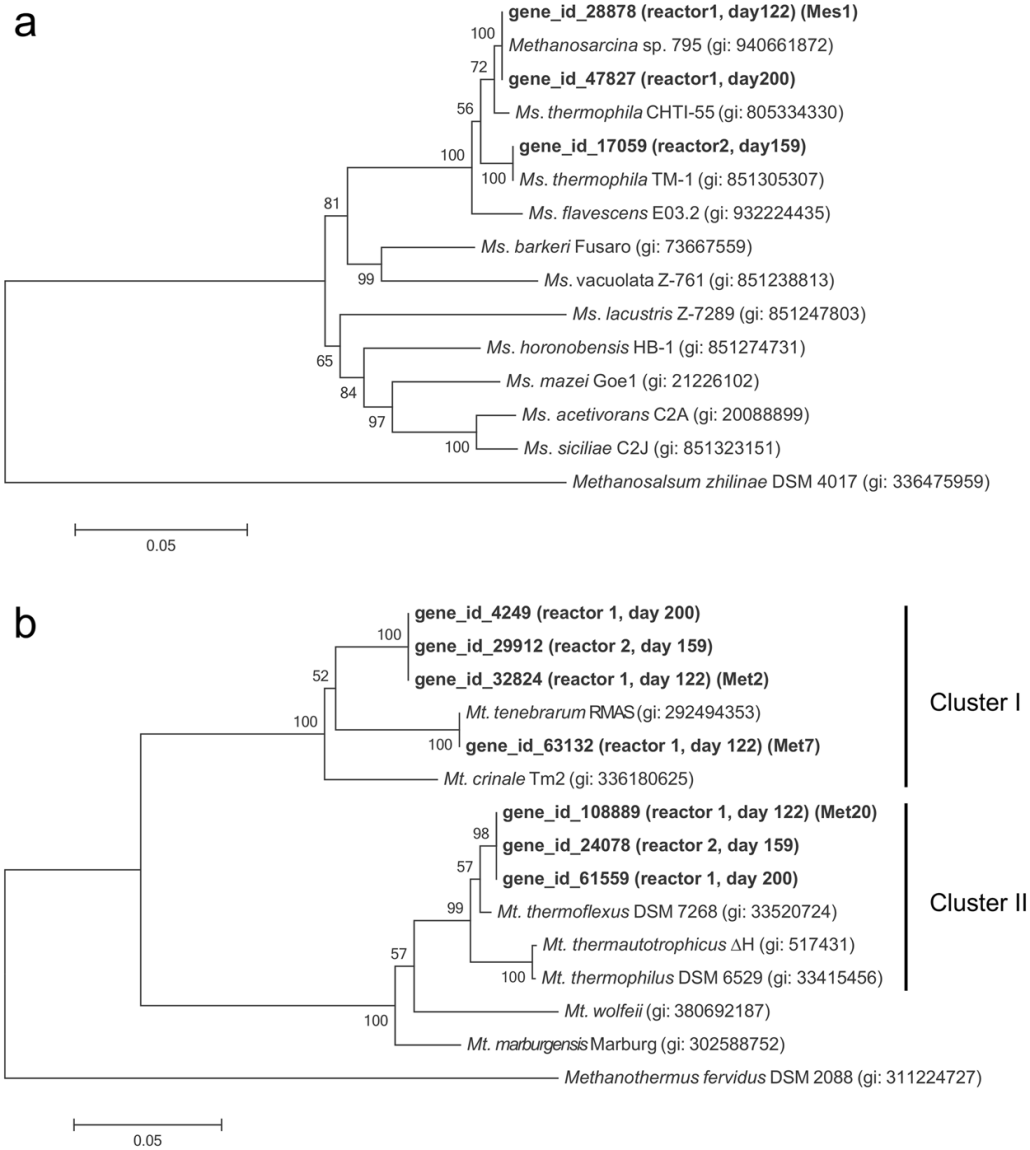
Neighbor-joining trees based on nucleotide sequences of *mcrA* genes showing phylogenetic relationships in the genus *Methanosarcina* (**a**) and *Methanothermobacter* (**b**). Bootstrap values (100 trials, only >50 are shown) are indicated at branching points. The bar indicates 5% sequence divergence. GI numbers are shown in parentheses.

To phylogenetically assign methanogens that occurred in the thermophilic digesters, we analyzed genes for methyl coenzyme-M reductase (*mcrA*) that have been used for the classification of methanogens^29, 30^. To this end, metagenomes extracted from BFs in reactor 1 (on days 122 and 200) and reactor 2 (on day 159) were shotgun-sequenced, and obtained reads were assembled to construct contigs. Sequencing and assembly data are summarized in Supplementary Table S2. Genes encoding McrA were extracted from the contigs and subjected to phylogenetic analyses. In this analysis, we detected only a few *mcrA* genes whose host methanogens were affiliated with the genera *Methanosarcina* and *Methanothermobacter* (Fig. 2a and b).

The *mcrA* phylogenetic tree constructed for *Methanosarcina* (Fig. 2a) showed that these reactors harbored methanogens affiliated with *Methanosarcina* (*Ms*.) *thermophila*, an acetoclastic methanogen frequently found in thermophilic anaerobic digesters^31, 32^. Two *mcrA* genes detected in reactor 1 (on days 122 and 200) were both identical to that of *Methanosarcina* sp. OTU 795 that was recently detected in an enrichment culture from an acetate-fed thermophilic digester in Canada^33^, and they were also very closely related to that of *Ms*. *thermophila* CHTI-55 isolated in France^34^. The *mcrA* sequence detected in reactor 2 was identical to that of *Ms*. *thermophila* TM-1 isolated in the USA^35, 36^. The fact that close relatives of *Ms*. *thermophila* have been widely detected from thermophilic anaerobic digesters all over the world suggest that this taxon ubiquitously plays an important role in these digesters regardless of geographical locations.

The *mcrA* tree for *Methanothermobacter* (Fig. 2b) indicated that this genus is divided into two clusters (named clusters I and II), and the present study detected *mcrA* genes grouped into both clusters. Cluster-I *Methanothermobacter* methanogens were closely related to *Methanothermobacter* (*Mt*.) *tenebrarum* RMAS^37^ and *Mt*. *crinale* Tm2^38^, which are thermophilic hydrogenotrophic methanogens isolated from a natural gas field water and oil reservoir sand, respectively. These two strains are reported to be non-autotrophic hydrogenotrophic methanogens that had been enriched and isolated in media containing acetate as a growth factor^37, 38^. It is therefore hypothesized that cluster-I *Methanothermobacter* methanogens, including those detected in our reactors, require acetate for growth and/or their growth is stimulated by acetate supplied to their habitats. On the other hand, cluster-II *Methanothermobacter* methanogens were closely related to autotrophic members within this genus, such as *Mt*. *thermoflexus* DSM 7268 and *Mt*. *thermautotrophicus* ∆H^39, 40^.

**Figure 3:**
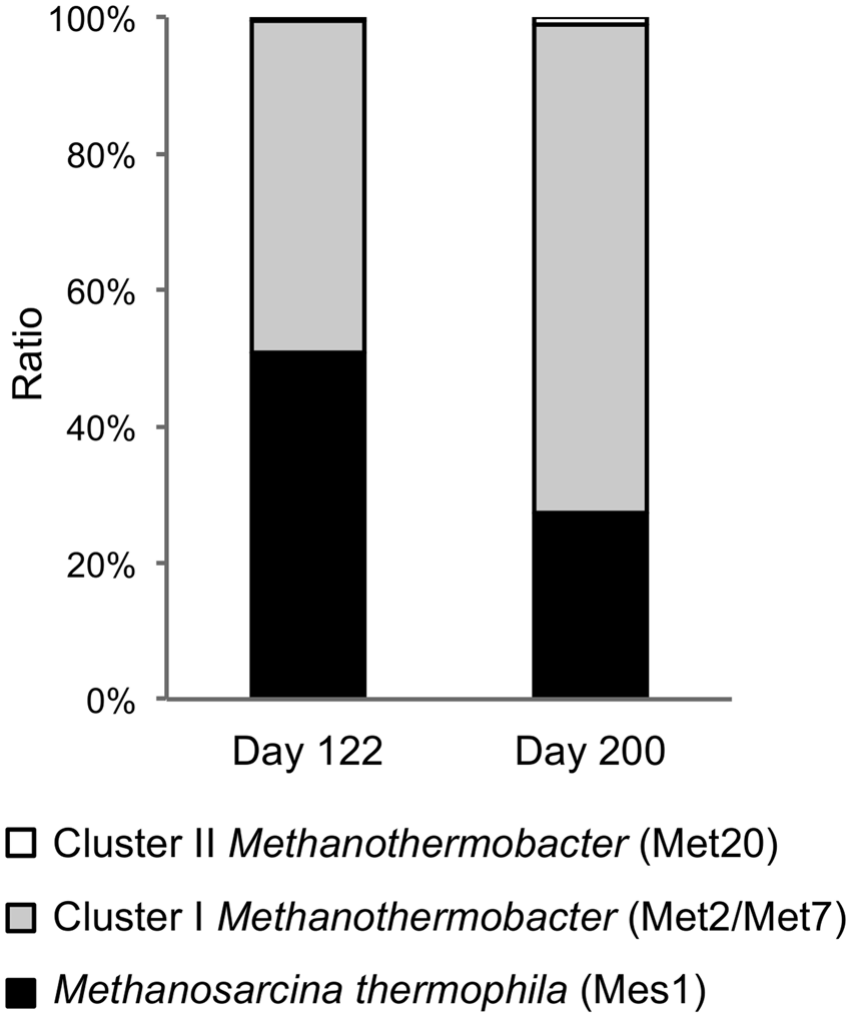
Relative abundances of methanogens in reactor 1 based on RPKM values for *mcrA* genes.

Abundance ratios of methanogens in reactor 1 as estimated from RPKM values for *mcrA* genes (Fig. 3) showed that cluster-II *Methanothermobacter* was limited (0.36% and 0.96% of the total methanogens on days 122 and 200, respectively) in the digesters, while methanogens belonging to *Ms*. *thermophila* and cluster-I *Methanothermobacter* were predominantly present. We therefore hypothesized that methanogens that can grow heterotrophically using acetate as a carbon source, such as acetoclastic *Methanosarcina* and non-autotrophic members of *Methanothermobacter*, are advantageous over autotrophic methanogens in anaerobic digesters that contain high concentrations of organics.

### Genome binning and reconstruction

**Figure 4:**
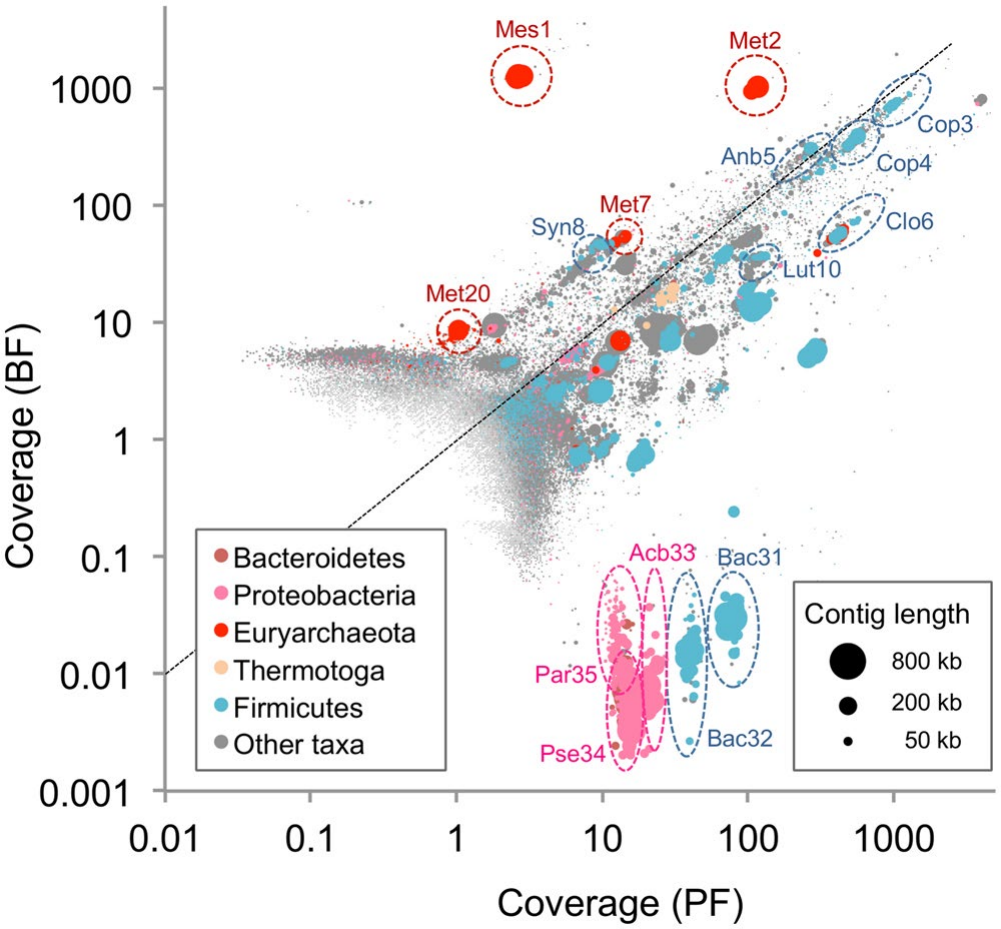
Differential coverage plot for assembled contigs. DNA reads from BF and PF samples were assembled together, and the coverage values of these reads were plotted for each contig. Contigs were phylogenetically classified at the phylum level using PhyloPythiaS^86^. Contig lengths correspond to bubble sizes. Representative bin-genomes are indicated by dotted ellipses.

To characterize genomic and metabolic features of the major members of the methanogenic consortia, we reconstructed individual population genomes (bin-genomes) from metagenome contigs. In this analysis, we used contigs assembled from reactor 1 on day 122 to construct representative bin-genomes, since all major methanogens of interest were included in this sample. Differential coverage binning of the contigs was conducted using coverage values for BF and PF reads (Fig. 4), and subsequent curation of binned contigs generated 39 high-quality bin-genomes (an average completeness value of over 92%), including two enclosed genomes for the dominant methanogens (designated Mes1 and Met2) and two draft genomes for relatively minor methanogens (Met7 and Met20) (Table 1 and Supplementary Table S3). Table 1 shows that the enclosed Mes1 and Met2 bin-genomes do not have the complete set of universal single-copy genes for archaea^41, 42^. However, they are considered to be complete, since the complete genome of *Ms*. *thermophila* TM-1 also does not have the complete set (98% in the completeness value; Supplementary Data 1). The bin-genomes were taxonomically assigned on the basis of 16S rRNA genes using the RDP classifier^43^. The taxonomic positions of the four reconstructed methanogens are presented in Supplementary Fig. S3, showing that the *mcrA* phylogeny (Fig. 2) is in good agreement with that based on 16S rRNA genes.

**Table 1:** Major bin-genomes reconstructed from the PF and BF samples in reactor 1.

Taxonomy^a^	Bin ID	Frequency^b^ (%)		Length (Mbp)	No. of CDS	No. of contigs	Estimated Completeness^c^ (%)
		PF	BF				
Methanogenic archaea							
*Methanosarcina*	Mes1	0.1	33.9	3.20	2772	1	99
*Methanothermobacter*	Met2	3.3	23.9	1.52	1604	1	98
*Methanothermobacter*	Met7	0.4	1.1	1.50	1651	8	97
*Methanothermobacter*	Met20	0.03	0.2	1.56	1678	25	99
Bacteria							
*Coprothermobacter*	Cop3	25.6	15.0	0.97	1146	226	91
*Coprothermobacter*	Cop4	14.8	8.2	1.31	1500	266	84
*Anaerobaculum*	Anb5	6.9	6.4	1.50	1538	147	95
*Clostridium* III	Clo6	11.3	1.3	3.40	3229	199	92
*Syntrophaceticus*	Syn8	0.3	1.0	2.04	2075	116	95
*Clostridia*	Clo9	2.7	1.2	3.20	2848	222	94
*Lutispora*	Lut10	3.2	0.8	2.11	2086	192	93

^a^Taxonomic positions assigned by RDP classifier with a confidence threshold of 80%.

^b^Calculated based on RPKM values of PF and BF reads for each bin-genome.

^c^Estimated based on the frequency of universal single-copy genes (136 genes for archaea and 105 genes for bacteria) in each bin-genome.

Relative frequencies of the reconstructed bin-genomes (Supplementary Table S3) show that the top 10 most abundant bin-genomes in BF accounted for 92.8% of the total BF population. The differential coverage plot for reconstructing bin-genomes (Fig. 4) and their relative frequencies (Table 1) show that Mes1 and Met2, which are closely related to *Ms*. *thermophila* and *Mt*. *tenebrarum*, respectively, were dominantly present in BF, accounting for 33.9% and 23.9%, respectively, of the total BF population. This result corresponds to those obtained by the phylogenetic analysis of 16S rRNA gene amplicons (Fig. 1b) and the abundance analysis of the *mcrA* genes (Fig. 3). Although two other methanogens, Met7 and Met20, were also enriched in BF, their relative abundance was less than 5% and 1%, respectively, of that of Met2 (Table 1). Met7 is closely related to Met2 (100% identity in 16S rRNA gene sequence) and other non-autotrophic members of *Methanothermobacter*, such as *Mt*. *tenebrarum*. On the other hand, Met20 is closely related to autotrophic *Methanothermobacter* methanogens, such as *Mt*. *thermautotrophicus* ∆H (Fig. 2b and Supplementary Fig. S3b). These results also support the hypothesis that autotrophic methanogens that do not utilize organic substrates are minor components of microbiomes occurring in anaerobic digesters that contain high concentrations of organics.

### Bin-genomes of syntrophic and fermentative bacteria

In addition to the bin-genomes for the four methanogens, the analysis reconstructed 35 bacterial draft genomes, including those for syntrophic and fermentative bacteria affiliated with *Syntrophaceticus* (Syn8), *Coprothermobacter* (Cop3 and Cop4), *Anaerobaculum* (Anb5), and *Clostridiales* (e.g., Clo6 and Lut10) (Table 1 and Supplementary Table S3). Syn8 was abundantly present in BF and relatively closely related to *Syntrophaceticus schinkii*, a syntrophic acetate-oxidizing bacterium isolated from a mesophilic anaerobic digester^44^. It is therefore likely that Syn8 was syntrophically associated with Met2 and other hydrogenotrophic methanogens by producing hydrogen from acetate (i.e., SAO). Cop3 and Cop4 were closely related to *Coprothermobacter proteolyticus*, an anaerobic proteolytic bacterium that is frequently found in thermophilic digesters^45, 46^, suggesting that these bacteria also contributed to syntrophic methanogenesis by producing hydrogen from organics that were present in the reactor (e.g., proteins released from dead cells). Clo6 and Lut10 were the most closely related to *Clostridium* (*Ruminiclostridium*) *thermocellum* and *Lutispora thermophila*, respectively, which are both thermophilic fermentative bacteria that utilize yeast extract as a growth factor^47, 48^. It is therefore conceivable that yeast extract contained in the growth media stimulated the growth of these fermentative bacteria. In addition to these anaerobic bacteria, the analysis also revealed that several putative aerobic bacteria, belonging to the genera *Bacillus* (Bac31 and Bac32), *Acinetobacter* (Acb33), *Pseudomonas* (Pse34), and *Paracoccus* (Par35), were specifically present in PF (Fig. 4 and Supplementary Table S3). We assume that these bacteria grew by consuming contaminated oxygen in PF and contributed to maintaining anaerobic conditions in the reactor.

### Genomic features of dominant methanogens

**Figure 5:**
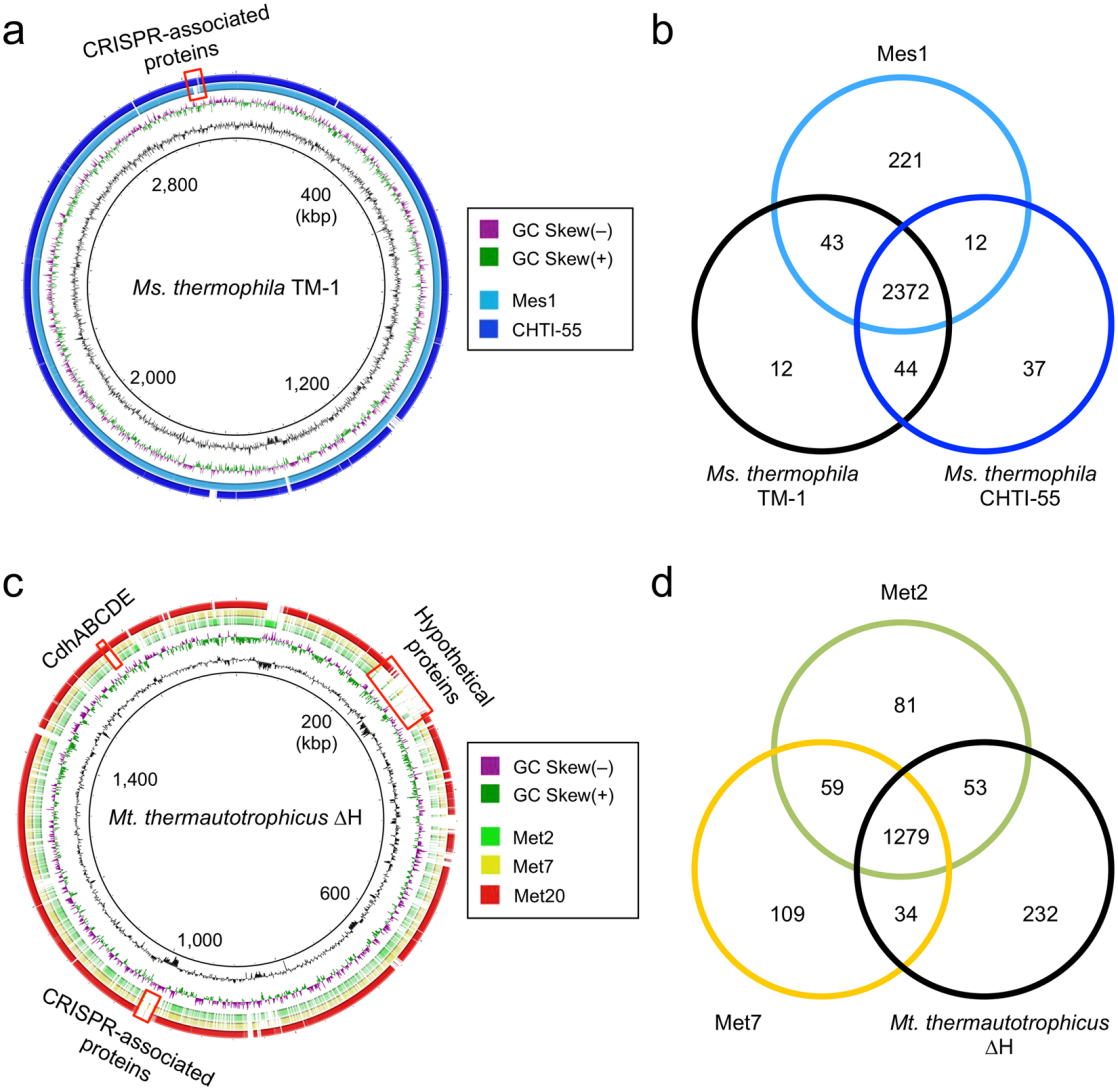
Comparative genomics of reconstructed *Methanosarcina* (**a**,**b**) and *Methanothermobacter* (**c**,**d**) strains. (**a**,**c**) Overall genome comparisons with closely related isolates using BRIG. The homology regions with the genomes of *Ms*. *thermophila* TM-1 (BLASTN e-value ≤ 1e-9) and *Mt*. *thermautotrophicus* ∆H (e-value ≤ 1e-2) are indicated by colors. The first (inner-most) and second circles show the GC contents and GC-skew, respectively. The locations of some genes of interest are indicated by red boxes. (**b**,**d**) Venn diagrams showing peculiar and shared CDSs coded in *Methanosarcina* (**b**) and *Methanothermobacter* (**d**). Redundant genes were excluded from the analysis.

We were interested in characterizing genomic and metabolic features of the dominant methanogens (i.e., Mes1 and Met2) to gain insights into how they abundantly grew in the anaerobic digesters. To this end, we comparatively analyzed the reconstructed bin-genomes of Mes1, Met2, Met7, and Met20 together with genomes of phylogenetically related isolates as references. The *in silico* DNA-DNA hybridization (DDH) analysis^49^ revealed that the reconstructed Mes1 genome was almost identical to those of *Ms*. *thermophila* TM-1 and *Ms*. *thermophila* CHTI-55 (98.7% and 96.8% identical in the DDH values, respectively). Genome-wide comparisons of Mes1, *Ms*. *thermophila* TM-1, and *Ms*. *thermophila* CHTI-55 using BLAST Ring Image Generator (BRIG)^50^ also show that these three strains are highly similar in their genome structures (Fig. 5a and Supplementary Fig. S4a). This notion is also supported by the synteny-plot analysis (Supplementary Fig. S5). These results indicate that *Ms*. *thermophila* is ubiquitously present in thermophilic anaerobic digesters without substantial changes in genome sequences. It is conceivable that this methanogen is highly evolved to adapt to thermophilic anaerobic digesters.

Comparisons of CDSs among the three *Ms*. *thermophila* methanogens by the BLAST-based bidirectional best-hit (BBH) analysis^51, 52^ extracted 314 genes that are differentially present in these methanogens (Fig. 5b). Among them, we focused on 221 genes that are present only in Mes1 (Supplementary Data 2) and 44 genes that are present in the two reference strains but lost from Mes1 (Supplementary Data 3). Although many of these genes encode hypothetical proteins without functional annotations, we found that some of them are CRISPR (clustered regularly interspaced short palindromic repeats)-associated genes (representatives are illustrated in Fig. 5a and Supplementary Fig. S4a). The CRISPR locus uniquely found in Mes1 (Supplementary Fig. S4a) contained different spacer sequences from those found in the reference strains (data not shown). This indicates that these methanogens have different histories of phage infection, regardless of phylogenetic similarities. We also found that several genes related to biofilm formation, such as genes involved in extracellular polysaccharide biosynthesis, are present only in Mes1 (Supplementary Fig. S4a and Supplementary Data 2). Given that the two reference *Ms*. *thermophila* strains were isolated from completely mixed digesters^34, 35^, it is likely that these genes (those uniquely found in Mes1) are related to the ability of this strain to form biofilm on support media in packed-bed reactors, and their products contribute to preventing cells from washout during high flow-rate operation.

Genome comparisons were also performed for the *Methanothermobacter* bin-genomes, Met2, Met7, and Met20. In this analysis, we used the genome of *Mt*. *thermautotrophicus* ∆H as the reference, since genome information of closely related *Methanothermobacter* species, such as *Mt*. *tenebrarum* and *Mt*. *crinale*, is not currently available, and strain ∆H is one of the most extensively characterized hydrogenotrophic methanogens in terms of physiological features and catabolic pathways^53, 54^. The DDH values of Met2, Met7, and Met20 to ∆H were 13.5%, 13.2%, and 86.5%, respectively, while the DDH value between Met2 and Met7 was 54.9%. These results confirm that the *Methanothermobacter* strains dominantly present in the reactors (i.e., Met2 and Met7) are taxonomically distinct from autotrophic members of this genus (e.g., strain ∆H). The DDH analysis also suggests that Met2 and Met7 may represent different species, even though their 16S rRNA gene sequences are identical (Supplementary Fig. S3b). We comparatively analyzed the genomic features of Met2 and Met7 by the BBH analysis with ∆H as the reference and extracted 249 genes unique in Met2 and/or Met7 but lost from ∆H and 232 genes present in ∆H but lost from the two reconstructed methanogens (Fig. 5d). As is the case for Mes1, genes differentially present in these *Methanothermobacter* methanogens included those encoding hypothetical proteins and CRISPR-associated proteins (Fig. 5c; Supplementary Data 4 to 7).

**Figure 6:**
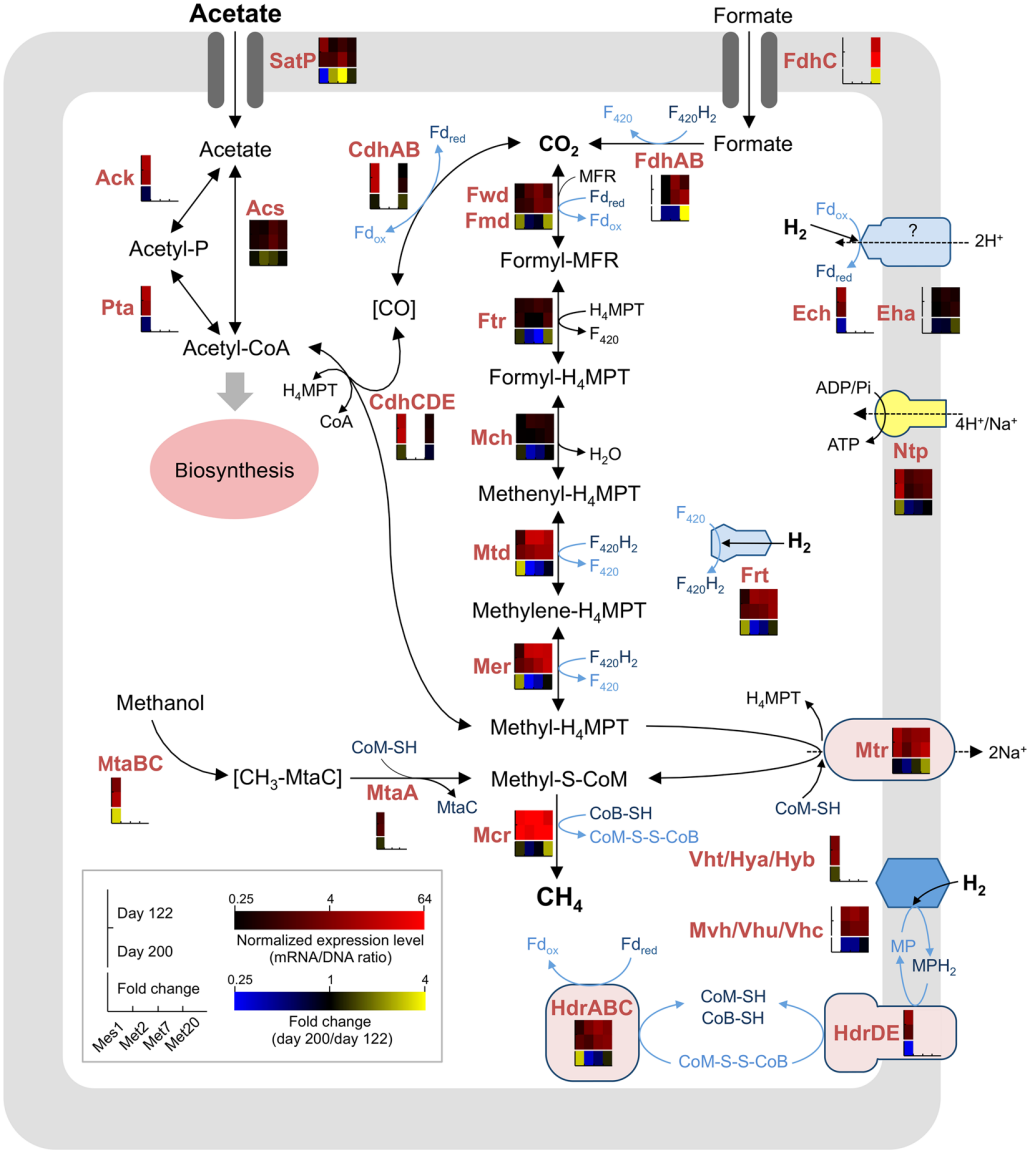
Reconstructed pathways for methanogenesis and associated metabolism in the four methanogens and their gene-expression profiles. Normalized expression levels (mRNA/DNA) and fold changes in the expression level (days 122/day 200) are shown as heatmaps. For an enzyme encoded by multiple subunit genes, average values for each gene are shown. Genes absent from each bin-genome are shown as blanks in heatmap boxes. The pathways are depicted according to previous reports^56, 57, 75, 87–89^. Abbreviations: Fd_ox_/Fd_red_, oxidized and reduced ferredoxin; MP/MPH_2_, oxidized and reduced methanophenazine; CoB-SH, coenzyme B; CoM-SH, coenzyme M; CoM-S-S-CoB, mixed disulfide of CoM-SH and CoB-SH; F_420_/F_420_H_2_, oxidized and reduced Factor 420; H_4_MPT, tetrahydromethanopterin; Fwd, tungsten formylmethanofuran dehydrogenase; Fmd, molybdenum formylmethanofuran dehydrogenase; Ftr, formylmethanofuran:H_4_MPT formyltransferase; Mch, methenyl-H_4_MPT cyclohydrolase; Mtd, F_420_-dependent methylene-H_4_MPT dehydrogenase; Mer, methylene-H_4_MPT reductase; Mtr, methyl-H_4_MPT: coenzyme M methyltransferase; Mcr, methyl-coenzyme M reductase; Hdr, heterodisulfide reductase; Ech, energy-converting hydrogenase; Frh, F_420_-reducing hydrogenase; Mvh/Vhu/Vhc/Vht/Hya/Hyb, non F_420_-reducing hydrogenase; AcsS, acetyl-CoA synthetase; Ack, acetate kinase; Pta, phosphotransacetylase; Cdh, CO dehydrogenase/acetyl-CoA synthase; MtaBC, methanol: 5-hydroxybenzimidazolylcobamide Co-methyltransferase; MtaA, methylcobalamin:coenzyme M methyltranferase; Ntp; proton or sodium-translocating ATPase.

The BBH analysis also revealed that Met2 and Met7 lack the gene cluster encoding the CO dehydrogenase/acetyl-CoA synthase complex (CdhABCDE; Supplementary Data 7), although these genes were present in ∆H and Met20 (Fig. 5c), as well as many other genome-sequenced methanogens. The Cdh complex is required for interconversion among CO_2_, acetyl-CoA, and methyltetrahydromethanopterin (Methyl-H_4_MPT; see Fig. 6) and is the sole system for carbon fixation in *Mt*. *thermautotrophicus* ∆H. We therefore concluded that Met2 and Met7 are unable to assimilate CO_2_ and also unable to utilize acetate as a substrate for methanogenesis. This finding supports the idea that they are non-autotrophic hydrogenotrophic methanogens that require acetate as a carbon source and utilize hydrogen as an energy source. Interestingly, several hydrogenotrophic methanogens belonging to the orders *Methanomicrobiales*, *Methanocellales*, and *Methanobacteriales* lack the Cdh complex^55^. Among them, three mesophilic hydrogenotrophic methanogens, *Methanosphaera stadtmanae*, *Methanocella paludicola*, and *Methanobrevibacter ruminantium*, isolated from human feces, rice paddy-field soil, and bovine rumen contents, respectively, are reported to require acetate for their growth^56–58^. These facts suggest that non-autotrophic hydrogenotrophic methanogens are widely distributed in organic-rich methanogenic environments.

### Reconstruction of methanogenic pathways

To characterize catabolic pathways in the methanogens present in BF, we reconstructed their methanogenesis pathways on the basis of their coding sequence (CDS) information annotated by the KEGG Automatic Annotation Server (KAAS)^59^ and BLAST search^60^ against the NCBI nr database (Supplementary Data 8 to 12). For comparison, we also analyzed the methanogenesis pathways of *Ms*. *thermophila* TM-1 and *Mt*. *thermautotrophicus* ∆H using the same procedures (Supplementary Data 12). The reconstructed metabolic pathways (Fig. 6) indicate that the pathways for acetoclastic and hydrogenotrophic methanogenesis are completely conserved in the reconstructed *Methanosarcina* Mes1 and *Methanothermobacter* Met2, Met7, and Met20. However, we also found that Mes1 and *Ms*. *thermophila* TM-1 lack formate dehydrogenases (Supplementary Data 12), indicating that Mes1 is unable to utilize formate as a methanogenic substrate, as is reported for isolated *Ms*. *thermophila* strains^34, 35^ and other *Methanosarcina* species^61^. Similar to many other hydrogenotrophic methanogens, Met2, Met7, Met20 were found to possess the gene(s) encoding putative formate dehydrogenase (*fdhA* and *fdhB*; Supplementary Data 12). However, since the *fdhC* gene, which encodes a formate transporter conserved in *Methanothermobacter* sp. CaT2 and other formate-ulilizing hydrogenotrophic methanogens^62^, is not conserved in Met2 and Met7 as well as non-formate utilizing *Mt*. *thermautotrophicus* ∆H^40^, it is likely that these methanogens are unable to utilize formate. In support of this speculation, *Mt*. *tenebrarum* and *Mt*. *crinale*, which are closely related to Met2 and Met7, are also reported to be unable to grow on formate^37, 38^. On the other hand, the *fdhC* gene is conserved in Met20 (Supplementary Data 12), suggesting that this methanogen is capable of utilizing formate. It is therefore conceivable that formate availability is an important factor for the survival of this methanogen in anaerobic digesters.

### Mutation accumulation during reactor operation

To evaluate whether the reconstructed methanogens were stably maintained in the reactor after they underwent the long-term and high-OLR operation, we analyzed genetic mutations accumulated in the methanogens using the DNA reads sampled on day 200. These DNA reads were mapped into the bin-genomes of Mes1, Met2, Met7, and Met20 that were constructed using the reads sampled on day 122. We found that only three to 20 mutations, including several single nucleotide polymorphisms (SNPs), were introduced into Mes1, Met2, or Met7 (Supplementary Table S4), indicating that these three methanogens were maintained without substantial changes in their genome sequences after they were exposed to the high-OLR condition. However, the analysis also showed that the minor methanogen Met20 underwent a relatively large number of mutations, including 1621 SNPs (Supplementary Table S4). This result suggests that there exists the genetic diversity below the species or subspecies level (microdiversity) within the Met20 population, and a variant of Met20 that adapted to the high OLR conditions preferentially grew before day 200. Similar observations that the microdiversity exists among bacterial and archaeal groups present at low abundances have been reported for other microbial ecosystems^63, 64^.

### Transcriptional dynamics in the methanogenesis pathways

To gain insights into how methanogens respond to changes in operational conditions of anaerobic digesters, metatranscriptomic analyses were conducted based on the reconstructed bin-genomes for the four methanogens. Particular attention was paid to effects of OLR on the methanogenesis pathways, and we comparatively analyzed transcriptomic profiles of these methanogens on days 122 and 200 (Fig. 6 and Supplementary Data 8 to 11).

It was found that, in these four methanogens, the genes encoding key enzymes for acetoclastic and/or hydrogenotrophic methanogenesis, i.e., Mtd, Mer, Mtr, and Mcr, were abundantly expressed both on days 122 and 200 (Fig. 6), indicating that methanogenesis by these methanogens were active under both conditions. The abundant expression of these genes under methanogenic conditions has also been reported in previous studies^65–68^. The *mrtBDGA* operon, which encodes methyl-coenzyme M reductase II (MRII) found in *Mt*. *thermautotrophicus* ∆H^69, 70^, is conserved only in Met20; however, these genes were only slightly expressed both on days 122 and 200 and did not exhibit marked expression changes between the two conditions (Supplementary Data 11). Given that transcription of the *mrt* genes are up-regulated when excess H_2_ is supplied^69^, it is conceivable that sufficient amounts of H_2_ were not supplied to Met20 even under the high OLR condition. In Met20, however, the *fdhCAB* genes involved in formate utilization were abundantly expressed, and their expression levels were increased on day 200. This result suggested that formate served as an important methanogenic substrate for this methanogen, particularly under the high OLR condition.

Interestingly, we found that Mes1 exhibited increased expression levels of the *fwd*, *mtd*, *mer*, and *frt* genes, which are involved in hydrogenotrophic methanogenesis, on day 200 as compared to those on day 122. This result suggested that Mes1 preferentially utilizes the hydrogenotrophic pathway rather than the acetoclastic pathway under high OLR conditions. Increased expression levels of genes involved in hydrogenotrophic methanogenesis on day 200 were also observed for Met20 (Fig. 6). Together with the increased population ratios of the *Methanothermobacter* methanogens (Figs 1b and 3), it is suggested that the hydrogenotrophic methanogenesis coupled to SAO favorably operates for methanogenesis from acetate under high OLR conditions. This possibility is also suggested in previous studies^10, 71–74^, whereas the present study suggested that this shift in the methanogenesis pathway involves regulation at the transcriptional level. In addition, we found that expression of the *mtaBC* genes involved in methanogenesis from methanol increased in Mes1 on day 200, suggesting that this methanogen also activated the methanol-dependent methanogenesis pathway under the high OLR condition. Further studies, including metabolic flux analysis using ^13^C-labeled acetate, are needed to deepen our understanding of ecophysiological and molecular mechanisms underlying the shift in the methanogenesis pathways in response to OLR.

The metatranscriptomic analysis also revealed that the *satP* gene, which encodes a putative acetate transporter conserved in prokaryotes^75^, was expressed in Met2 and Met7 at higher levels on day 200 than those on day 122, whereas the expression of this gene was not substantially different in Met20 between these two days. These results suggested that the increased acetate supply under the high OLR condition promoted acetate uptake activity of Met2 and Met7 cells and promoted their heterotrophic growth, although autotrophic Met20 cells did not directly respond to this environmental stimulus. In Mes1, the expression of the *satP* gene was lower on day 200 than that on day 122. We assume that this transcriptional shift also contributed to the switching from acetoclastic to hydrogenotrophic methanogenesis under the high OLR condition. Taken together, these gene expression profiles observed in the four methanogens suggest that they have the ability to adapt to environmental changes in anaerobic digesters by regulating expression of genes related to methanogenesis at the transcriptional level.

## Conclusions

The present study suggested that heterotrophic methanogens, i.e., acetoclastic and non-autotrophic hydrogenotrophic methanogens, are predominant in anaerobic digesters. Comparative genomics demonstrated that the genome of the dominant acetoclastic methanogen (Mes1) was almost identical to that of *Ms*. *thermophila* TM-1, whereas the analysis also revealed that several genes related to biofilm formation were uniquely present in the Mes1 genome. On the other hand, the major *Methanothermobacter* methanogen (Met2) lacked the genes for acetyl-CoA synthesis from CO_2_ (*cdhABCDE*), suggesting that this organism utilized acetate as the carbon source in association with hydrogenotrophic methanogenesis for energy conservation. To our knowledge, this is the first report describing the genome of non-autotrophic members of the genus *Methanothermobacter*. Since the autotrophic hydrogenotrophic methanogen Met20 was present in the anaerobic digesters in much less abundance than Met2, we suggested that the heterotrophic lifestyle confers an ecological advantage on methanogens for thriving in anaerobic digesters. Furthermore, we also suggest that methanogens have well-developed regulatory mechanisms for controlling their metabolism in response to changes in nutritional situations. In future studies, we will address molecular mechanisms in methanogens for sensing external stimuli and ecological significance of heterotrophic methanogens in diverse environments.

## Methods

### Bioreactor operation

Two laboratory-scale packed-bed reactors with similar configurations (reactors 1 and 2) were independently operated. A 1-L capacity jar fermentor was packed with support media composed of 300 cm^2^ (for reactor 1: 300 × 100 × 10 mm) or 251 cm^2^ (for reactor 2: 251 × 100 × 10 mm) of carbon fiber textiles (CFT; SPR25075PE; GRP, Osaka, Japan) and filled with 700 mL of modified medium A^76^ containing 8.7 g/L acetic acid and 0.5 g/L yeast extract as the sole sources of carbon and energy. Yeast extract was added to stabilize methanogenic consortia. Each reactor was initially seeded with anaerobic sludge sampled from a commercial thermophilic methane fermentation reactor and purged with pure nitrogen gas to remove headspace oxygen. The medium was pumped into the top of the reactor with a peristaltic pump, and the effluent was discharged through the overflow line. The contents of the reactor were moderately mixed by circulating the fermentation liquid using a stirrer. The temperature in the reactor was maintained at 55 °C during operation. Biogas produced in the reactor was collected from a Tedlar bag connected to the biogas line. OLR and HRT of the reactors were controlled by changing the flow rate of the medium.

### Chemical analysis

Methane, H_2_, and CO_2_ in the biogas were measured using a gas chromatograph (GC-14A; Shimadzu, Tokyo, Japan) equipped with a thermal conductivity detector (TCD). The total chemical oxygen demands (COD) of the medium and effluent were determined using a dichromate method according to the Japanese Industrial Standard (JIS) K-1012. The amount of acetate, propionate, and some other organic acids in fermentation liquid were measured using a high-performance liquid chromatography organic acid analysis system (LC-20A, Shimadzu) according to the manufacturer’s instructions. The pH of the fermentation liquid was monitored using a pH meter (LAQUA twin B-712, Horiba, Tokyo, Japan). The protein content of PF and BF was determined using the B-PER II bacterial protein extraction reagent (Pierce, Rockford, IL, USA) and Micro bicinchoninic acid (BCA) protein assay kit (Pierce) according to the manufacturer’s instructions.

### DNA and RNA extraction

Samples for nucleic acid extraction were collected from the reactors when methane was stably produced in the steady state of operation at applied HRT, i.e., after operation for 122 days (OLR: 5.9 g/L-reactor/day) and 200 days (OLR: 37.2 g/L-reactor/day) in reactor 1 and after operation for 159 days (OLR: 21.1 g/L-reactor/day) in reactor 2. Total DNA and RNA was extracted from the cell pellet collected from 42 mL of the fermentation liquid (for PF) and 1 cm^2^ pieces of the CFT support media (for BF). DNA was extracted using the FastDNA SPIN Kit for Soil (MP Biomedicals, Solon, OH, USA) according to the manufacturer’s instructions. DNA quality was assessed by agarose gel electrophoresis, spectrophotometric analysis, and the Quant-iT dsDNA BR assay kit (Invitrogen, Carlsbad, CA, USA). RNA was extracted using TRIzol reagent (Invitrogen) and was purified using an RNeasy Mini Kit and RNase-Free DNase Set (Qiagen, Valencia, CA, USA). The quality of extracted RNA was evaluated using an Agilent 2100 Bioanalyzer with RNA 6000 Pico reagents and RNA Pico Chips (Agilent Technologies, Santa Clara, CA, USA).

### PCR amplification and sequencing of 16S rRNA gene fragments

PCR amplification of 16S rRNA gene fragments (V4 region) from the metagenomic DNA was performed using primers ad-tag-515F (5′-CGTATCGCCTCCCTCGCGCCATCAGXXXXXXGTGCCAGCMGCCGCGGTAA-3′) and ad-tag-806R (5′-CTATGCGCCTTGCCAGCCCGCTCAGGGACTACHVGGGTWTCTAAT-3′), in which the underlined sequences were adaptors for pyrosequencing and XXXXXX was an arbitrary tag sequence for sample identification. PCR conditions were described elsewhere^77^. Amplicons were purified using a QIAquick PCR purification kit (Qiagen) and subjected to pyrosequencing using a Genome Sequencer FLX system. Ten- to forty-thousand reads were obtained for each sample, and phylogenetic analyses were conducted using the Silva rRNA database (http://www.arb-silva.de/).

### Metagenomic DNA and RNA sequencing, mapping and assembly

Approximately 5 µg of quality-checked DNA was used to construct paired-end and fragmented libraries and sequenced using the HiSeq 2000 sequencing system (Illumina, San Diego, CA, USA) as described elsewhere^52^. Reads with quality scores were trimmed using CLC Genomics Workbench version 6.5.1 (CLC Bio Japan, Tokyo, Japan) with default parameters. Quality-trimmed reads obtained from the BF and PF samples were mixed and assembled into contigs with scaffolding based on paired-end information using CLC Genomics Workbench with a kmer size of 53 and bubble length of 800 bp. Contigs over 500 bp in length were used for subsequent gene prediction and binning analysis. RNA sequencing was performed using the TruSeq RNA Sample Preparation Kit V2 (Illumina) and HiSeq 2000 sequencing system. Prior to cDNA library preparation, rRNA was removed from the total RNA samples using the Ribo-Zero rRNA Removal kit for bacteria (Epicentre, Wisconsin, USA) according to the manufacturer’s instructions. RPKM (reads per kilobase per million mapped reads) values were calculated by mapping DNA or RNA reads to assembled sequences (bin-genomes or ORFs) using CLC Genomics Workbench with default settings, except for the use of 0.7 as the minimum length and 0.97 as the minimum similarity fractions. A normalized gene expression level for each gene (mRNA/DNA ratio) was calculated by dividing the mRNA-RPKM for each ORF by the DNA-RPKM for the ORF. A fold change in expression of each gene on days 122 and 200 was calculated as a ratio between normalized gene expression levels under the two conditions. Values were visualized as heatmaps using the MultiExperiment Viewer (MeV) software^78^.

### Gene prediction and annotation

Coding sequences (CDS) in contigs were predicted using MetaGeneMark^79^. Gene identification and annotation were performed by the KEGG Automatic Annotation Server (KAAS)^59^ using the single-directional best-hit method and cutoff bit score of 45. 16S rRNA genes in contigs were annotated by MiGAP (http://www.migap.org) and taxonomically assigned by the RDP classifier^43^ with a confidence threshold of 80. Alignment of 16S rRNA and *mcrA* gene sequences and construction of their neighbor-joining trees were conducted using the MEGA program ver. 6.06^80^.

### Genome binning and reconstruction

Contig clustering and draft genome reconstruction were conducted using a multi-step process, including differential coverage binning and tetranucleotide frequency analysis, according to methods described previously^81, 82^. The bin-genomes of the four methanogens were further refined by connecting the selected contigs using the mapping information of paired-end reads. The connections of the contigs were checked using the Cytoscape software version 2.8.3^83^, and associated contigs were assembled using Genome Finishing Module in CLC Genomics Workbench. The genome of Mes1 was constructed by aligning contigs using the genome of *Methanosarcina thermophila* TM-1 as the reference. The completeness of bin-genomes was assessed by core-gene analysis^42^ using 107 marker genes for *Bacteria* ^84^ and 137 marker genes for *Archaea* ^42^. The marker gene list for *Archaea* was constructed based on the comparison of 99 archaeal genomes using a method described previously^41^.

### Comparative genomics

Overall genome comparisons between the reconstructed methanogens and closely related isolates were performed using BLAST Ring Image Generator (BRIG)^50^ with e-value cut-offs of 1e-9 (Mes1 vs. *Methanosarcina thermophila* TM-1) and 1e-2 (Met2, Met7, and Met20 vs. *Methanothermobacter thermautotrophicus* ∆H). Peculiar and shared CDSs in different genomes were extracted by the bidirectional best-hit (BBH) analysis (Overbeek *et al*.^51^) using the BLASTP program^60^ according to a method described previously^52^. Redundant functional genes were excluded from the analysis. Synteny dot-plot analysis was performed using the r2cat program^85^.

### Nucleotide variant detection for methanogen bin-genomes

Nucleotide variants (polymorphisms) in the methanogen sequences were detected using Basic Variant Detection tool in CLC Genomics Workbench with default settings. DNA reads obtained from BFs on day 122 and day 200 were mapped into each methanogen bin-genome constructed from the DNA reads on day 122, and nucleotide variants (i.e., single- and multiple-nucleotide polymorphisms, deletion, and insertion) that were specifically detected on the day 200 sequences were extracted. Nucleotide variants commonly detected on the day 122 and day 200 sequences were regarded as repeated sequences with small variations in each bin-genome (e.g., inverted and tandem repeats in transposons), and they were excluded from the analysis.

## Electronic supplementary material

Supplementary Information

Supplementary Data 1

Supplementary Data 2

Supplementary Data 3

Supplementary Data 4

Supplementary Data 5

Supplementary Data 6

Supplementary Data 7

Supplementary Data 8

Supplementary Data 9

Supplementary Data 10

Supplementary Data 11

Supplementary Data 12
