# Supplementary material for: Non-autotrophic methanogens dominate in anaerobic digesters
Source: Sci Rep. 2017 May 4;7:1510. doi: 10.1038/s41598-017-01752-x (PMC5431450; doi:10.1038/s41598-017-01752-x)
Supplement: Supplementary file 1 — Supplementary Information [file 41598_2017_1752_MOESM1_ESM.pdf]

Non-autotrophic methanogens dominate in anaerobic digesters

- Supplementary Information -

Atsushi Kouzuma*, Maho Tsutsumi, Shun'ichi Ishii, Yoshiyuki Ueno, Takashi Abe, and Kazuya Watanabe

*Corresponding author
E-mail: akouzuma@toyaku.ac.jp

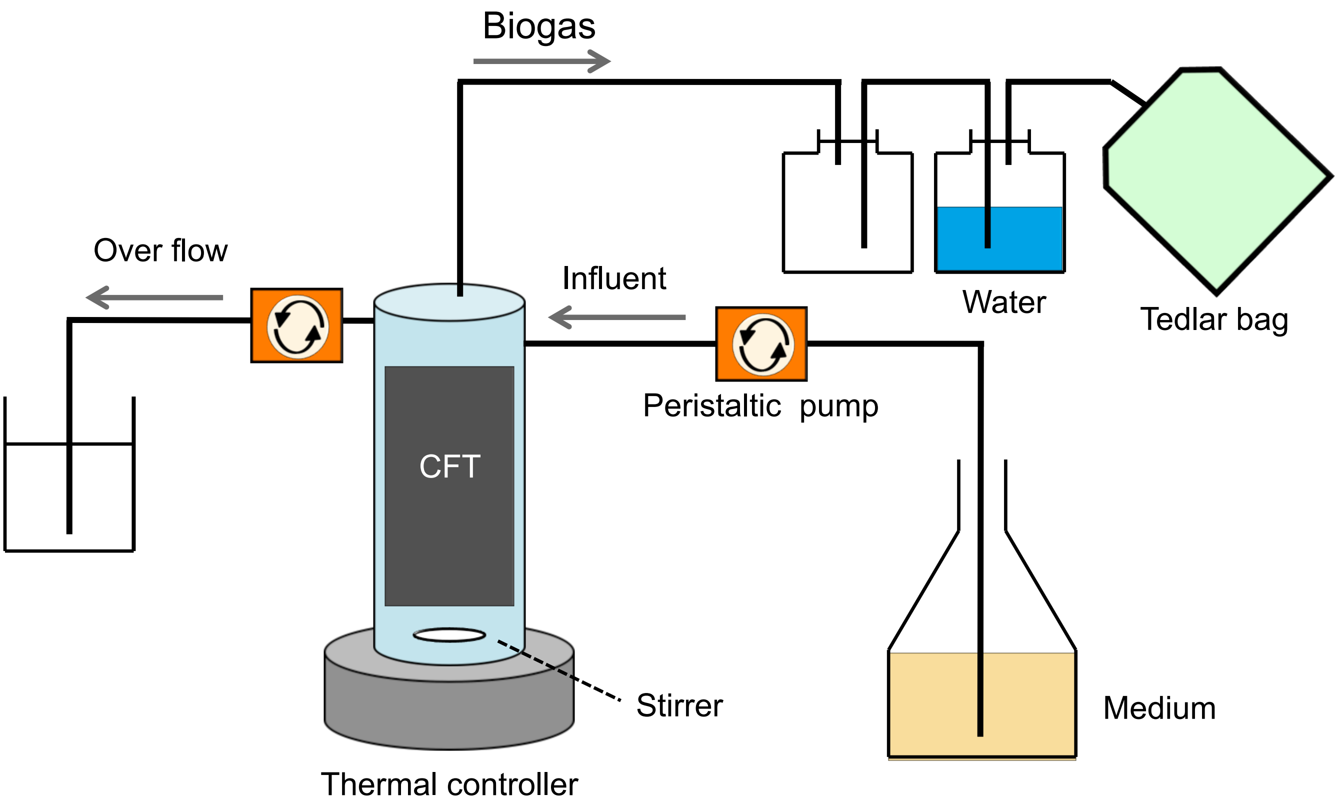

Figure S1. Configuration of reactors used in this study.

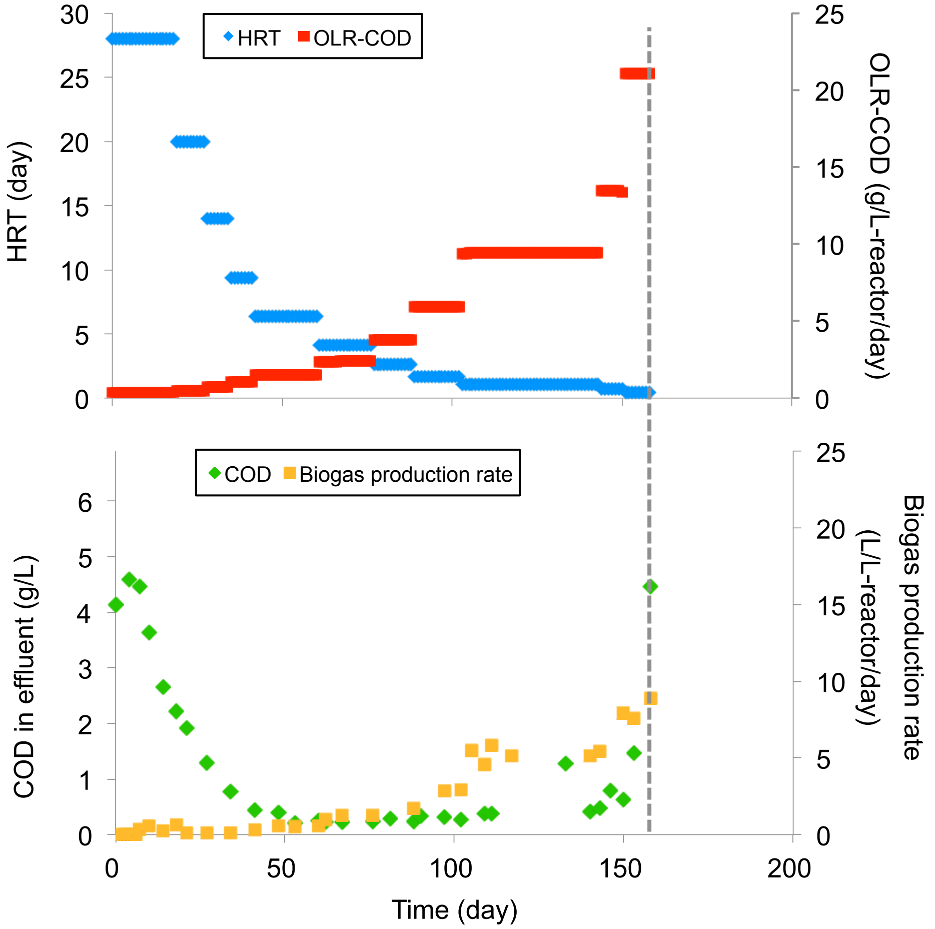

Figure S2. Time course of OLR, HRT, COD in effluent, and biogas production rates in reactor 2. The sample collection date for the metagenomic and metatranscriptomic analysis (day 159) is indicated by a dotted line.

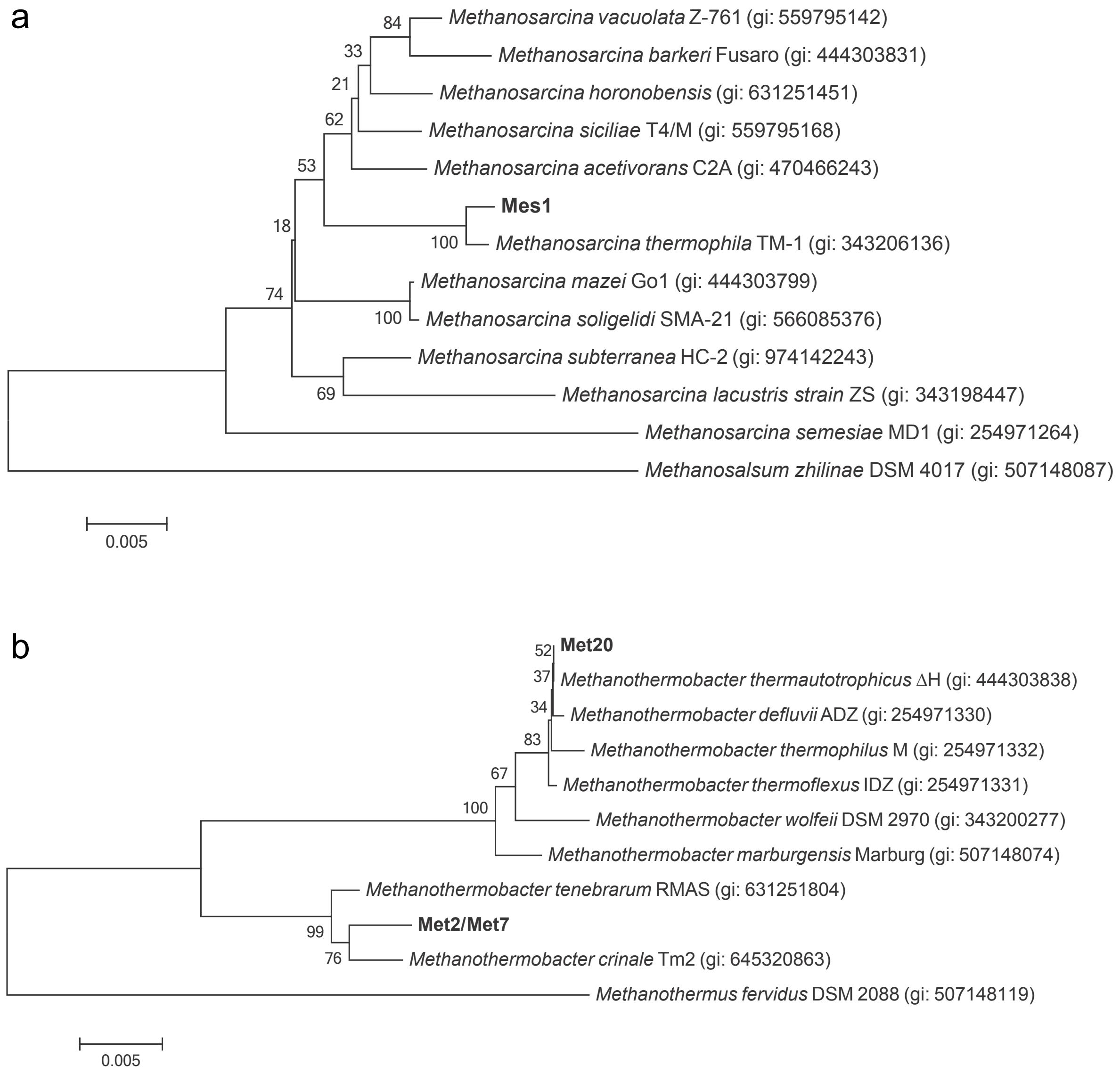

Figure S3. Neighbor-joining tree based on nucleotide sequences of 16S rRNA genes showing phylogenetic relationships in the genus *Methanosarcina* (a) and *Methanothermobacter* (b). Bootstrap values (100 trials, only >50 are shown) are indicated at branching points. The bar indicates 0.5% sequence divergence. GI numbers are shown in parentheses.

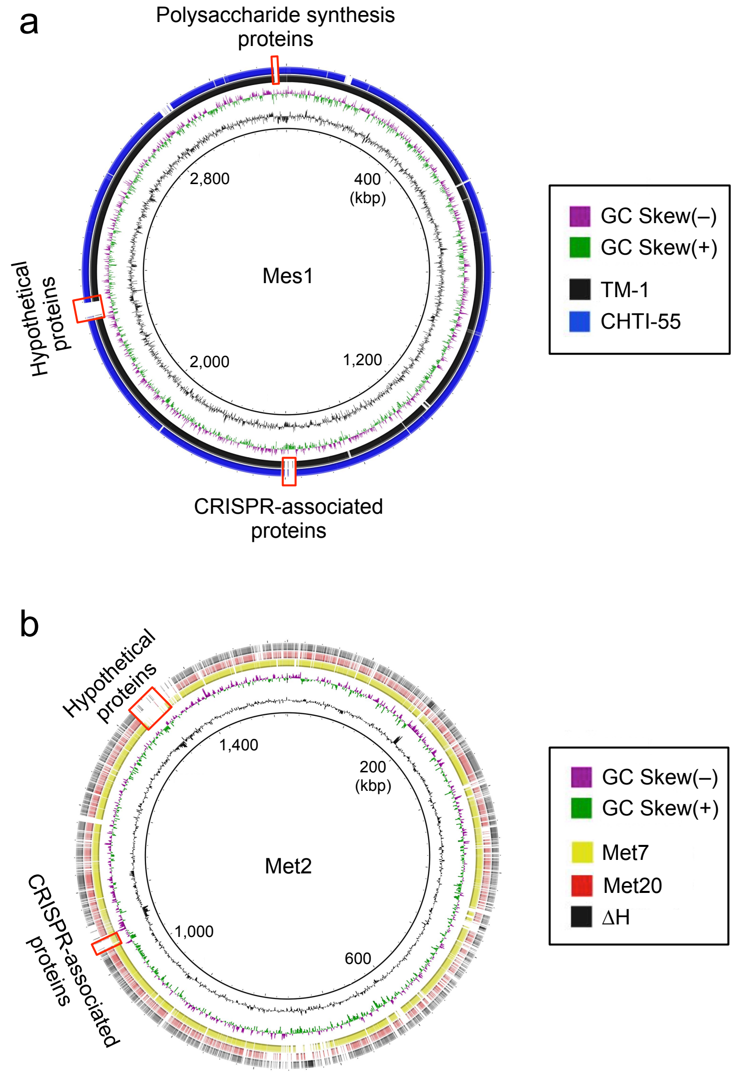

Figure S4. BRIG images using Mes1 (a) and Met2 (b) genomes as references. The homology regions with the genomes of Mes1 (BLASTN e-value ≤1e-9) and Met2 (e-value ≤1e-2) are indicated by colors. The first (inner-most) and second circles show the GC content and GC-skew, respectively. The locations of some genes of interest are indicated by red boxes.

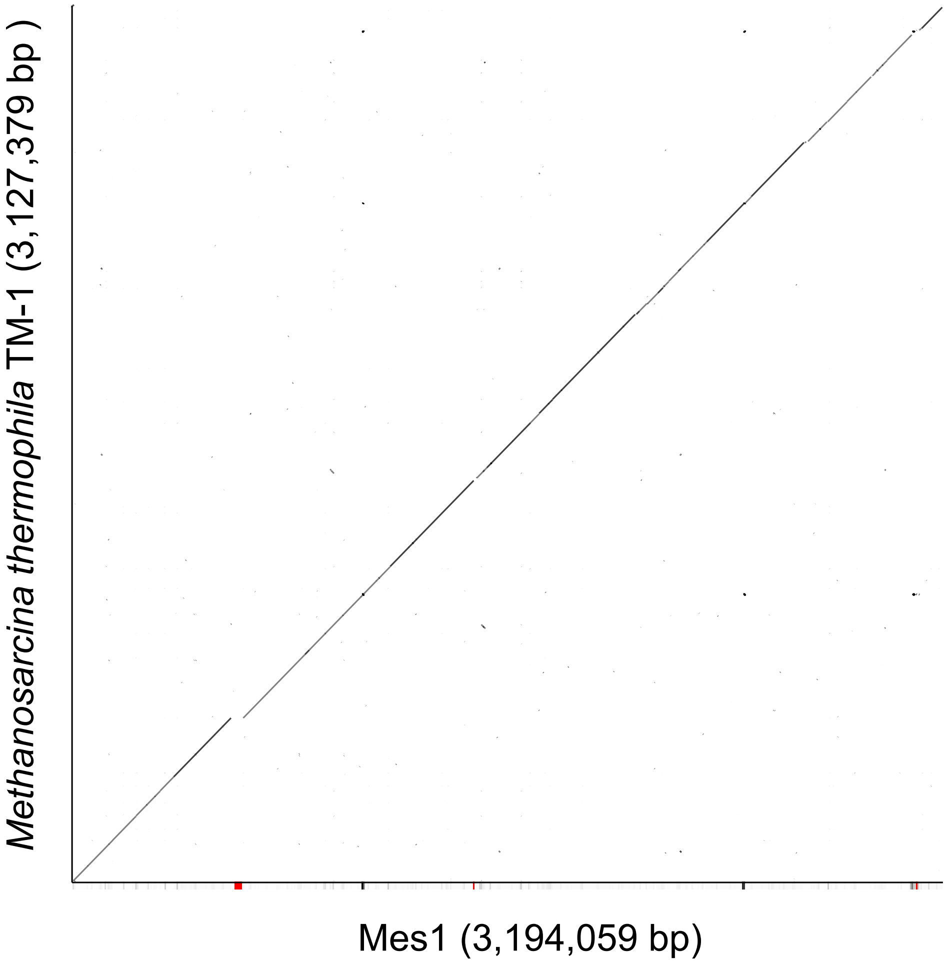

Figure S5. Synteny dot plot between Mes1 and *Methanosarcina thermophila* TM-1 genomes. Gray dots represent the homologous DNA region between the two methanogens.

Table S1. Performance parameters for reactor 1 and reactor 2.

Parameter[a]		Reactor 1		Reactor 2
		Day 122	Day 200	Day 159
HRT (day)		1.7	0.3	0.5
OLR (g/L-reactor/day)	COD	5.9	37.2	21.1
OLR (g/L-reactor/day)	Acetate	4.7	29.2	17.9
Biogas production rate (L/L-reactor/day)		3.45	22.65	8.89
Effluent	COD (mg/L)	142	1129	2971
	Acetate (mg/L)	23.6	479	1723
	Propionate (mg/L)	9.05	65.2	63.2
	pH	6.9	6.7	6.4
Removal ratio (%)	COD	97.2	88.7	69.8
	Acetate	99.2	93.9	79.3
Methane production yield (%)[b]		100	113	100
Biomass (mg/mL)[c]	PF	0.0522	0.0281	0.359
	BF	1.29	26.9	23.0

[a]Data shown are average of values collected during each steady state of operation period.

[b]The ratio of experimental methane production yield to theoretical methane production yield calculated based on COD removal ratio. The theoretical methane potential was calculated from 350 mLCH_4/gCOD. Methane gas concentration in the biogas was assumed as 58%.

[c]The protein concentration in microbial cells collected from each fraction.

Table S2. Summary of metagenomic sequencing and assembly.

Sample name	Description	No. of reads[a]		Total read length (bp)	No. of contigs[b]	Total contig length (bp)[b]	N50 (bp)[b]	No. of CDS[b]
		PF	BF					
R1_D122_DNA	DNA from reactor 1 on day 122	189,730,176	188,992,618	37,521,286,131	68,129	189,840,872	14,030	227,393
R1_D200_DNA	DNA from reactor 1 on day 200	159635878	146,710,738	30,218,344,676	63,397	169,742,023	13,548	208,966
R2_D159_DNA	DNA from reactor 2 on day 159	52,127,542	61,078,732	11,334,532,371	22,754	110,369,745	29,150	127,381
R1_D122_RNA	RNA from reactor 1 on day 122	-	197,969,722	19,994,941,922	-	-	-	-
R1_D200_RNA	RNA from reactor 1 on day 200	-	55,769,551	5,632,724,651	-	-	-	-

[a]The number of DNA reads represents the number of DNA reads after being quality-trimmed.

[b]Calculated for contigs more than 500 bp in length.

Table S3. Full list of bin-genomes reconstructed in this study.

Bin ID	Taxonomy[a]	Frequency[b] (%)		Length (Mbp)	CDS	No. of contigs	Estimated Completeness[c] (%)
		PF	BF				
Mes1	*Methanosarcina*	0.1	33.9	3.20	2772	1	99
Met2	*Methanothermobacter*	3.3	23.9	1.52	1604	1	98
Cop3	*Coprothermobacter*	25.6	15.0	0.97	1146	226	91
Cop4	*Coprothermobacter*	14.8	8.2	1.31	1500	266	84
Anb5	*Anaerobaculum*	6.9	6.4	1.50	1538	147	95
Clo6	*Clostridium* III	11.3	1.3	3.40	3229	199	92
Clo9	*Clostridia*	2.7	1.2	3.20	2848	222	94
Met7	*Methanothermobacter*	0.4	1.1	1.50	1651	8	97
Syn8	*Syntrophaceticus*	0.3	1.0	2.04	2075	116	95
Rum11	*Ruminococcaceae*	1.8	0.8	2.38	2343	189	94
Lut10	*Lutispora*	3.2	0.8	2.11	2086	192	93
Bct12	*Bacteria*	0.4	0.7	2.43	2223	53	91
Atr13	*Atribacteria*	0.2	0.7	1.64	1677	115	91
Fir16	*Firmicutes*	2.7	0.4	2.24	2209	87	96
Unc14	-	0.5	0.4	1.84	1784	443	26
Pet15	*Petrotoga*	0.8	0.4	1.95	1798	76	98
Eth17	*Ethanoligenens*	3.1	0.3	1.86	1708	26	96
Thd18	*Thermodesulfovibrio*	0.0	0.2	1.75	1781	51	99
Fir25	*Firmicutes*	0.8	0.2	2.45	2151	94	94
Rum19	*Ruminococcaceae*	0.7	0.2	2.18	1951	10	97
Met20	*Methanothermobacter*	0.0	0.2	1.56	1678	25	99
Fir23	*Firmicutes*	1.3	0.2	2.32	2056	23	94
Unc30	-	0.8	0.2	2.65	2704	58	99
Bct26	*Bacteria*	0.4	0.2	2.18	2109	54	97
Def24	*Defluviitalea*	7.7	0.1	3.24	3099	46	94
Bcr29	*Bacteroidetes*	0.2	0.1	2.12	1857	213	96
Unc27	-	0.3	0.1	3.99	3435	165	92
Bct36	*Bacteria*	0.3	0.1	2.65	2541	230	88
Unc39	-	0.2	0.1	2.22	2163	137	55
Tep37	*Tepidanaerobacter*	0.3	0.1	3.19	3321	73	96
Unc28	-	0.2	0.1	2.85	2376	108	94
Unc21	-	0.3	0.0	2.92	2662	165	96
Unc22	-	0.5	0.0	4.01	3372	105	95
Bac31	*Bacillus*	2.1	0.0	3.50	3663	46	99
Bac32	*Bacillus*	1.1	0.0	3.85	3999	66	98
Par35	*Paracoccus*	0.3	0.0	3.49	3612	276	99
Acb33	*Acinetobacter*	0.6	0.0	2.73	2641	25	99
Ptm38	*Petrimonas*	0.4	0.0	3.65	3108	57	97
Pse34	*Pseudomonas*	0.4	0.0	6.42	6053	90	98

[a]Taxonomic positions assigned by RDP classifier with a confidence threshold of 80%. Unclassified ("Unc") bin-genomes (i.e., Unc14, Unc21, Unc22, Unc27, Unc28, Unc30, and Unc38) are not classified because *16S* rRNA genes were not binned for these bin-genomes.

[b]Calculated based on RPKM values of PF and BF reads for each bin-genome.

[c]Estimated based on the frequency of universal single-copy genes (136 genes for archaea and 105 genes for bacteria) in each bin-genome.

Table S4. Nucleotide variation in reconstructed methanogen genomes between day 122 and day 200[a].

	SNV[b]	MNV[c]	Insertion	Deletion
Mes1	4	0	0	0
Met2	1	2	0	0
Met7	14	5	0	1
Met20	1621	30	23	14

[a]Values indicate the number of nucleotide variants detected by mapping the day 200 reads to each bin-genome constructed from the day 122 reads.

[b]Single nucleotide variants.

[c]Multiple nucleotide variants.
